# The Urinary Tract and the Catheter

**Published:** 1986-10

**Authors:** J. P. Mitchell


### THE URINARY TRACT AND THE CATHETER, INFECTION AND OTHER PROBLEMS

**Norman Slade and William A. Gillespie**
**John Wiley and Sons, Chichester**

It is a very great pleasure to see the numerous studies carried out by Norman Slade and William Gillespie on the subject of urinary tract infection collated in book form. Although this book is small it presents a wealth of knowledge gained from years of practical experience. The book is comfortable to read and the apparent simplicity is only possible because both authors have such a thorough and extensive knowledge of their subject.

At this time when we are being encouraged by the D.H.S.S. to practise every economy possible, the suggested rationalisation of catheter purchase by Supplies Officers must come as a very welcome thought to our newly appointed Director of Procurements for the D.H.S.S. Norman Slade's extrapolation of the cost of the stock of catheters held throughout the Hospitals in this country must surely indicate a ready means for substantial economies. On several occasions he implies the extravagent use of expensive catheters where much cheaper catheters would be perfectly adequate.

Most urologists will be very pleased to read the repeated condemnation of the catheter introducer in the hands of anyone who is not thoroughly experienced in urological procedures.

The only possible trivial criticism might be on the subject of liquid paraffin being injected down the balloon inflation channel to dissolve a balloon that won't deflate. The standard viscous liquid paraffin will not go down the inflation channel without excessive pressure but there is a very much thinner form of liquid paraffin that can be obtained for this by special request. However, in my experience if the bladder is empty and the balloon will not deflate, it is more effective to put standard liquid paraffin down the lumen of the catheter and into the bladder, where it will be in direct contact with the outside of the balloon. The balloon will then dissolve and rupture within forty minutes.

Apart from this minor criticism this book is an outstanding contribution to urological literature and should be read not only by urologists but also by junior staff, all of whom will at some time have to handle and deal with catheters. This book should also be of considerable help to nurses, nursing tutors and those who have to advise patients and their relatives on the management of catheters and the control of incontinence. In addition this book should be read by all who serve on control of infection committees. It is to be hoped that every hospital authority ensures that this book is available in the hospital library.

J. P. Mitchell

